# Preparedness to Combat Determinants of Underweight-Based Child Malnutrition in Flood-Affected Areas of Pakistan

**DOI:** 10.1155/2022/6464901

**Published:** 2022-01-25

**Authors:** Ijaz ul Haq, Xiang He, Abdul Majid Mujahid, Hira Ibrahim, Zafar Mehmood, Jahan Shah, Bilal Ahmed, Amjad Khan, Shahbaz Ahmad Zakki, Ihtisham Ul Haq, Muhammad Shahzad, Javed Muhammad, Jielian Xu, Saeed Ahmed, Muhammad Sohail, Jing Miao

**Affiliations:** ^1^Department of Public Health & Nutrition, The University of Haripur, Haripur, Khyber Pakhtunkhwa, Pakistan; ^2^Department of Clinical Nutrition, The Affiliated Jiangning Hospital of Nanjing Medical University, Nanjing, Jiangsu 211100, China; ^3^Basic Health Unit Daiwal, Tehsil/District Khushab, District Health Authority Khushab, Punjab, Pakistan; ^4^Jinnah Burn and Reconstructive Surgery Center Lahore, Pakistan; ^5^Department of Math's, Stats & Computer Science, The University of Agriculture Peshawar, Pakistan; ^6^Department of Social Medicine and Health Education, School of Public Health, Nanjing Medical University, Jiangsu Province, China; ^7^School of Pharmacy, Nanjing Medical University, Nanjing, Jiangsu 211166, China; ^8^Department of Microbiology, The University of Haripur, Haripur, Khyber Pakhtunkhwa, Pakistan; ^9^Department of Biological Sciences, National University of Medical Sciences, Rawalpindi, Pakistan; ^10^School of Pharmacy, Key Laboratory of Molecular Pharmacology and Drug Evaluation, Yantai University, China; ^11^Department of Clinical Nutrition, Nanjing Tongren Hospital, School of Medicine, Southeast University, Nanjing, Jiangsu 211100, China

## Abstract

**Aims:**

Floods badly impact the food and nutrition security in developing countries. The role of the government and the impact of floods on the underweight status of children in the affected areas is not clear. We aimed to find the determinants of underweight in flood-affected areas of Khyber Pakhtunkhwa, Pakistan.

**Methods:**

We used a multistage sampling technique and selected 656 households during in the flood-affected areas of Pakistan. Data were collected in the three most affected districts. A validated questionnaire was used to find socioeconomic and demographic information, hygiene, and sanitation information. We used logistic regression to find the determinants of underweight, controlling for confounders.

**Results:**

The prevalence of global malnutrition based on underweight was 25.2%. The prevalence of underweight was higher in young age mothers (40.6%), younger age children (71.4%), large family size (28.4%), joint family (27%), and no toilet facility (28.9%). District Nowshera was at high risk of underweight based undernutrition, followed by district Charsadda compared to children belonging to Dera Ismail Khan. The significant risk factor that causes underweight was child lower age (*p* < 0.01), young age of mothers (*p* < 0.01), children access to unimproved water sources (*p* < 0.01), and location (districts) due to environmental and constant flood consequences (*p* < 0.01).

**Conclusion:**

In conclusion, risk factors of underweight should be appropriately targeted in the flood-hit areas of Pakistan. Governments should preallocate budgetary resources and enhance the emergency preparedness levels to facilitate the communities with flooding incidents and their aftermath in the shape of child underweight-based malnutrition.

## 1. Introduction

The nutritional status of children affected by flooding situations is studied in many parts of the world [1]. Malnutrition is a leading cause of morbidity and mortality in children and is widespread in developing countries [2], especially flood-affected areas [3]. Stunting is more common in disaster-prone areas across the world. Underweight and stunting are very common in flooded communities compared with nonflooded areas in the same country with similar lifestyles [4]. Floods have both, directly and indirectly, impact on the underweight status. The direct impact of floods is a shortage of food required for proper nutrition and disruption of essential food items supply [5]. Contrarily, the flood also results in loss of income, homelessness, food cooking, production, contamination, displacing the children, contamination of water, and food sources [6, 7].

Literature reveals strong linkages of flooding and the underweight situation of children in South Asian countries, including India and Pakistan [8]. Developing countries face challenges of the underweight and stunting situation resulting in flood situations. Cascading events in floods often bring diseases like typhoid, diarrhea, dengue, malaria that collectively contribute to underweight, stunting, and wasting conditions for the children in certain communities. Management of such events most often requires multisectoral coordination and collaborations by many of the government agencies. There are four important response features to the floods by the governments containing, (a) safety and security of the people and their property, (b) supply of food and water items, (c) public health safety and security to protect the health of all, especially children, and (d) attain normal social life [9–11].

The demographic settlements of communities in high-risk vulnerable areas are affected many times by flooding. Most of such societies belong to countries India, Pakistan, Nepal, and Bangladesh. Children who belong to those vulnerable communities are often found underweight and stunted [12]. The flood in 2010 in Pakistan was a major disaster that affected nearly 21 million people and 11000 villages. It impacted the health status of the people in many ways [13]. The flood brought many short-term and long-term consequences for the government of Pakistan. In this disaster, the government has to manage child health and underweight issues by considering the loss of safe food, eliminating standing crops, transportation of dietary items to affected areas, decontamination of water reservoirs, loss of livestock resulting in meat and milk scarcity, and essential medications.

Underweight is one of the essential anthropometric measures of observing child malnutrition and is a combined form of undernutrition that comprises both stunting and wasting. It is defined as the percentage of preschool and school-going children whose weight for age is below minus two standard deviations (moderate and severe underweight) and minus three standard deviations (severe underweight) from the median of the WHO Child Growth Standards [14]. Underweight is related to deprived physical stamina to work and a weak immune system to resist diseases. Moreover, the high prevalence of underweight monitors the continued existence of undernutrition during the crucial days of child growth.

UNICEF (2013) reported that in 2011, about 101 million children under five years of age, or approximately 16 percent of children, were underweight. The prevalence is highest in South Asia with 59 million underweight children, followed by sub-Saharan Africa has 30 million. Globally, the prevalence of underweight has been declined, from 25 percent in 1990 to 16 percent in 2011—a 37 percent reduction [15].

Since undernutrition, including underweight continues during child growth, it is necessary to investigate the disparity effect of underweight prevalence on diverse socioeconomic and demographic determinants. Also, it is necessary to state how the government should prepare and cope with the disaster situation and the aftermath of disasters in Pakistan. The current study aimed to find the determinants of underweight in preschool-going children (5-60 months) and school-going children (>5 to 12 years of age) in the flood-affected areas of Khyber Pakhtunkhwa, Pakistan. We used underweight as a vital index of the nutritional status of children.

## 2. Methods

The cross-sectional study design was employed for this study in flood-affected areas of Khyber Pakhtunkhwa, Pakistan. Data was collected in 656 preschools and school-going children (1-12 years) in three affected districts, Charsadda, Nowshehra, and Dera Ismail Khan, from June 2014 to June 2016. The standard formula (*n* = (1.96)^2^ × 0.309 (1 − 0.309)/(0.05)^2^ = 328) was used for sample size calculation. The final calculated sample size was *n* = 656, as estimated samples size “328” was multiplied with the assumed “2” design effect (DE).

Multistage and simple random sampling techniques were used to select tehsils and union councils (UCS) and subdivided according to villages in the selected districts. The trained personnel visited respondent's household at a specific interval. Children with chronic illnesses and those of nonconsenting parents were excluded from the research.

The University of Agriculture, Peshawar, Pakistan, ethical committee approved this research in accordance with the Declaration of Helsinki's guidelines (IRB#002). Written informed consent was obtained from the research respondents.

A rapid assessment field survey questionnaire was developed in English and local languages (Urdu and Pashto) and pretested on 40 samples. Questionnaires were modified according to the initial data. Data were collected by trained and experienced personnel.

Demographic and socioeconomic informations were collected, which included gender, child and maternal age, household status (internally displaced persons (IDPs) or host), family size (small = 5 members, medium = 5 − 10 members, large = >10 members), family type (joint or nuclear family), father and mother's occupation (full-time working, part-time working, not working), monthly income (Pakistani rupees), father and mother's education, quality of water (Improved sources and unimproved sources), and toilet facility (pit latrine, flush, no facility) at household.

Weight-for-age *Z*-score was used to evaluate underweight-based malnutrition for under-five children according to the WHO criterion [16–18]. For more than five years of children, NCHS references were used to find underweight status [19].

SPSS version 20.0 (SPSS, Chicago, IL, USA) was used for statistical analysis. Emergency Nutrition Assessment (http://www.scribd.com) software was used for the calculation of *z*-scores. We calculated odds ratio (OR) and 95% confidence intervals (CIs) for the strength of association with underweight. Binary and ordinal logistic regression was used to find the determinants of underweight in the flood-affected region. A two-tailed *p* < 0.05 was considered significant.

## 3. Results

Table 1 depicts the percentage distribution of underweight-based malnutrition in preschool and school-going children of the target population and its association with socioeconomic determinants. Prevalence of underweight decreased with child age (i.e., from 71.4% to 16.2%). The proportion of underweight is high among mothers aged 15-24 and 25-36 years (40.6% and 19.4%, respectively). The majority (24.5%) of hosted children are underweight. Children living in joint families (27.0%) are more likely to be underweight than a child living in individual families (20.3%). Regarding parental education, the prevalence of underweight is higher in children having illiterate fathers and mothers (24.8% and 26.6%, respectively). Underweight decreased with increasing income level (i.e., from 27.1% to 19.7%). More (32.5%) children are malnourished concerning water quality and do not have improved water access. A majority (29.3%) child are underweight, having no toilet facility in their households. The proportion of underweight is high (37.7%) in district Nowshehra compared to the others two districts Charsadda and Dera Ismail Khan. The factors child age, maternal age, household status, family type, mother education, water quality, toilet facility, and districts are significantly (*p* value < 0.05) associated with child underweight.

High underweight prevalence exists in preschool going children in the flood-hit areas of Khyber Pakhtunkhwa (Table 2), where approximately one in 4 children (25.2%; 20.6-30.4; 95% CI) have a global prevalence of underweight based malnutrition (<-2 *z*-score) in children of age 5-60 months. Whereas approximately one in 8 children (13.1%; 9.7-17.4; 95% CI) have a severe prevalence of underweight-based malnutrition (<-3 *z*-score) in children of age 5-60 months in flood-hit areas. It was also observed that the global prevalence of underweight in under five years of female children 26.4% (19.8-34.3; 95% CI) is greater than male children 24.1% (18.1-31.3; 95% CI). Similarly, prevalence of underweight based severe malnutrition in female children 15.7% (10.6-22.6; 95% CI), respectively, is high as compared to male children 13.1% (9.7-17.4; 95% CI) while prevalence of moderate malnutrition (<-2 *z*-score and ≥-3 *z*-score) was found low in female children 10.7% (6.6-16.9; 95% CI) as compared to male children 13.3% (8.9-19.5; 95% CI).

As shown in Table 3, among 656 children, the overall prevalence of underweight in the study sample is 153 (23.3%) and is higher 72 (25.3%) in girls than in boys 81 (21.8%). The study also revealed that 108 (16.5%) are moderately underweight, and 45 (6.9%) are severely underweight. Similarly, 60 (16.2%) boys and 48 (16.8%) girls are moderately underweight. At last, 21 (5.7%) boys and 24 (8.4%) girls are severely underweight.

Figure S1 (a) shows the parentage of underweight and normal children in each age group and tells that there is a decreasing trend of malnutrition with the age of children because there is a high prevalence of stunting at low age groups as compared to higher age group (>36 months). Figure S1 (b) expresses the percentage of underweight and normal children for each age group calculated within the target population's overall percentage of underweight and normal children.

Figure S2 shows that out of the total sample of size 656, 57.74% of boys are with normal weights, whereas 42.26% of girls are with normal weights. It is evident from the figure that male children are slightly more susceptible to underweight-based malnutrition than female children in the flood-affected areas of Khyber Pakhtunkhwa.

Figure S3 shows that out of the total observed underweight children in the study area district, Nowshera is contributing the highest prevalence of underweight based malnutrition (68.42%) followed by district Charsadda (46.63%) and Dera Ismail Khan is the lowest (1.974% only).

Figure S4 shows the pyramid for children age-stratified according to the districts. It has been shown that children belonging to district Nowshera were at high risk of underweight based undernutrition followed by district Charsadda, also susceptible to undernutrition based on underweight compared to children belonging to Dera Ismail Khan.

In the following Figure S5, the pyramid was constructed for children age-stratified according to gender. It showed that male children are slightly more susceptible to undernutrition based on underweight than female children.

The analysis in Table 4 revealed that children aged 1-12 months are 6.91 times (AOR = 6.91, 95% CI: 1.11, 13.08; *p* value = 0.038), children aged 12-24 months are 5.8 times (AOR = 5.89, 95% CI: 3.84, 12.01; *p* value < 0.001), and children aged 24-36 months are 4.78 times (AOR = 4.78, 95% CI: 2.62, 12.70; *p* value < 0.001) more at risk for underweight as compared children older age > 36 months. Children delivered by younger mothers, aged 15-24 years, are significantly 2.89 times more likely to be underweight than children delivered by older mothers above 34. Children who have no access to improved drinking water are 3.40 times more likely to be underweight than those who have access to improved drinking water (AOR = 3.40, 95% CI: 1.72, 6.71; *p* value < 0.001). Further analysis showed that children the belong to district Charsadda are 3.5 times more likely to be underweight (AOR = 3.58, 95% CI: 1.70, 6.74; *p* value < 0.001), and children from district Nowshera are 5.44 times more likely to be underweight (AOR = 5.44, 95% CI: 2.4, 9.91, *p* value < 0.001) as compared to children belongs to Dera Ismail Khan.

We have also applied the ordinal logistic regression model when the response variable is categorized into three levels: normal children, moderately prevalence of malnutrition, and severe prevalence of malnutrition (Table 5). The analysis showed almost similar significant socioeconomic determinants as in the binary logistic model, and it is found that child age, maternal age, family type, mother education, water quality, and toilet facility significant (*p* value < 0.05) risk factors of undernutrition.

## 4. Discussion

The prevalence of underweight was high in preschool and school-going children in the flood-hit areas of Khyber Pakhtunkhwa, Pakistan. It accounts for 25.2% of children aged 5-60 months, while 23.3% were underweight in children aged >5-12 years. The prevalence of underweight was higher in young age mothers (40.6%), younger age children (71.4%), large family size (28.4%), joint family (27%), and no toilet facility (28.9%). The findings also showed that the prevalence of underweight was less in children with literate parents than children with illiterate children. The significant risk factor that caused underweight was children of lower age, young mothers, children's access to unimproved water sources, and location (districts) due to environmental and constant flood consequences. We are the first study to report the determinants of underweight in the flood-hit areas of Pakistan. It is necessary to timely document the incidence of nutritional status reduce malnutrition. Timely documentation for improved communication is important [20].

In both age groups, female children were more likely to be underweight than boys in the flood-hit areas of KP. We collected data in Khyber Pakhtunkhwa, where the documented under five underweight according to the NNS 2018 was 23.1%, more than our presented data [19–21]. It means that flood has a negative impact on the prevalence of underweight-based malnutrition. We are the first to report underweight in more than five years' children in the flood-hit areas of Pakistan. Young women, infants, children, and adolescents are usually at high risk of malnutrition. Proper nutrition at an early age, including nutrition 1000 golden days, have long-term benefits to reduce this burden in later stages of life [14]. The high prevalence in >5-12 years children might be because the children are still growing but not getting enough calories and nutrients from a balanced diet during a disaster situation [22], which makes them underweight.

Due to environmental and constant floods, the locations are high and usually have a high prevalence of undernutrition. The impact of the flood on nutritional status is negative, which leads to poor nutritional status in under 5 and >5-12 year children [23, 24]. Therefore, the aftermath of floods should be seriously considered to eradicate malnutrition in children and their mothers [25].

Clean water is very important for every age group, including children. Safe drinking water and good sanitation and hygiene reduce the burden of undernutrition. Contaminated water and poor sanitation and hygiene status deteriorate child nutritional status causing undernutrition [26]. Unimproved water intake in flood-hit areas leads to stunting and MUAC-based undernutrition [23, 24]. Improved water and sanitation and toilet facilities lower mortality, diarrhea, and stunting in under-fie children [27].

Floods like disasters have the potential to cause food shortages and many nutritional issues in affected and sounding areas. Although modern transportation and information exchange means have changed the food distribution and consumption system, some communities have to rely on their crops, fruits, products, and indigenous nutritional items. At the same time, the vulnerable population living in hard areas and distanced populations could have the severe effect of flooding in terms of their nutritional needs. It impacts in two ways; the local crops eliminate, destroy much of saved items and affect production. Second, increased price of the food items and decreased the purchasing power of the people from flood-affected areas due to periodic unemployment and the burden of diseases.

The emergency response and mitigation structure in Pakistan are at a developing stage. To respond against the underweight issue of children due to flooding requires a comprehensive multisector emergency response and social development program involving all concerned organizations and separate relief funds. After the flooding events of 2001 and 2010 government of Pakistan implemented immediate emergency rescue activities, which saved lives and provided food essential for healthy life [28]. Envisioning underweight due to malnutrition in 2011, the government requested UNICEF, WFP, and other partners to provide nutritional supplements for children and pregnant women in affected areas [29].

The Government of Pakistan and international partners conducted “The Flood Affected Nutrition Survey (FANS)” to assess child nutrition status under five years of age in flood-affected communities [30]. The assessment provided the baseline information about malnutrition and loss of nutritional elements for the children and its potential consequences. The response to underweight children due to flooding is linked with federal and provincial government policies. Both governments are concerned with providing concrete steps with the support of national and international organizations to treat and manage underweight and malnutrition situations [31].

Interventions made to regulate the supplies of food items to the markets were not subsidized that each household could acquire daily. The government of Pakistan engaged many national and international NGOs in flood-affected areas and built coordination with the help of UNICEF and NDMA. Along with social and economic services, the organizations facilitated the affected families in providing ration, nutrition supplements, and cooked and uncooked food items for extended periods [32]. The main aim of the interventions was to eradicate the issue of malnutrition, including underweight among the affected children. Another program in collaboration with Action Against Hunger (ACF) was also launched with the name of Pakistan Emergency Food Security Alliance (PEFSA) program in 2011 in areas of Sindh province [33]. The output achieved by the program included timely identification and treatment of acute malnutrition and improved public awareness regarding underweight and the importance of nutrition during a flood situation.

The government of Pakistan, along with international partners, developed National Nutrition Cluster Preparedness and Response Plan to mainstream the activities and actions of partners for improving the nutritional statuses of vulnerable communities. The plan envisaged to emergencies exacerbate the underlying nutrition crisis in Pakistan [34]. 2010, 2011, and 2012 monsoon response the malnutrition for populations already suffering from emergencies of floods and monsoon flashings and had aftershocks. Despite many efforts, the targeted, planned step seems less likely to be taken to provide adequate relief for the children underweight in flood-hit areas. Studies reveal that Specialized Nutritious Foods (SNF) should be initiated to reduce malnutrition, including underweight situations. The government could start comprehensive targeted SNF provision interventions to all victim households in the affected area to support malnutrition issues. During last year, the Ministry of Poverty Alleviation and Social Protection planned the initiatives, including SNF implementation, to improve the nutritional status of children under two years and pregnant women [35]. Public emergency services such as ambulances would help mitigate services to prone areas, which may help reduce the disease burden [36].

There were certain limitations accounted for in this study. First, the study was a cross-sectional study that shows a causal impact. Second, this study was conducted after 4-5 years after the flood and reported this year. The reason is that we wanted to check the aftermath and long-term consequences of the flood. Flood is still a health concern affecting the nutritional status of children. Also, the role of government needed to be highlighted for preparedness in emergencies. Nevertheless, the current study is the first study highlighting the role of government and investigating the risk factors of underweight in the flood-affected areas of Pakistan.

## 5. Conclusion

In conclusion, risk factors of underweight, including child age, maternal age, family type, mother education, water quality, and toilet facility, should be appropriately targeted in the flood-hit areas of Pakistan. Floods often create long-term consequences for developing societies and marginalized people living in hard areas. Such disasters mostly bring social, economic, and health-related consequences for victims and government administrations. Such consequences of floods have been observed in the flood-affected areas of Pakistan. In Pakistan, it is uncommon to allocate the annual proportion of budgeting for food and nutrition security of the vulnerable population in case of flooding. During a disaster, the government has to engage the partners and funding agencies for preparedness and response to food and nutrition issues. Governments should preallocate budgetary resources and enhance the emergency preparedness levels to facilitate the communities with flooding incidents. SNF initiatives should also be included in the preparedness plans to facilitate the communities suffering from underweight child crises due to flooding and other similar hazards. Emergency services such as functional Rescue 1122 services may be helpful in pre and postdisaster time to reduce the disease burden.

## Figures and Tables

**Table 1:** Percentage distribution of underweight based malnutrition in preschool and school going children and its association with socioeconomic determinants.

Factors		Malnutrition based on underweight				Chi-square (*p* value)
		Normal		Underweight		
		>-2SD		<-2SD		
Gender	Male	290	78.2%	81	21.8%	1.23 (0.266)
	Female	213	74.7%	72	25.3%	
Age of child	1-12	2	28.6%	5	71.4%	85.5 (<0.01)
	13-24	19	42.2%	26	57.8%	
	25-36	29	46.8%	33	53.2%	
	>36	454	83.8%	88	16.2%	
Maternal age	15-24	98	59.4%	67	40.6%	38.95 (<0.01)
	25-34	208	80.6%	50	19.4%	
	>34	198	85.0%	35	15.0%	
Status of the household	Host	453	75.5%	147	24.5%	6.97 (0.008)
	IDP	51	91.1%	5	8.9%	
Family type	Joint family	206	73.0%	76	27.0%	3.96 (0.046)
	Individual family	298	79.7%	76	20.3%	
Family size	Small	134	80.7%	32	19.3%	5.08 (0.07)
	Medium	218	78.4%	60	21.6%	
	Large	151	71.6%	60	28.4%	
Father occupation	Not working	26	76.5%	8	23.5%	0.225 (0.894)
	Part time working	25	73.5%	9	26.5%	
	Full time working	453	77.0%	135	23.0%	
Mother occupation	House wife	480	77.2%	142	22.8%	0.794 (0.672)
	Part time working	10	71.4%	4	28.6%	
	Full time working	14	70.0%	6	30.0%	
Income	5000-10000	132	72.9%	49	27.1%	3.67 (0.16)
	10001-15000	144	75.4%	47	24.6%	
	>15000	228	80.3%	56	19.7%	
Father education	Literate	270	78.3%	75	21.7%	0.838 (0.36)
	Illiterate	234	75.2%	77	24.8%	
Mother education	Literate	231	81.3%	53	18.7%	5.71 (0.017)
	Illiterate	273	73.4%	99	26.6%	
Water quality	Improved	423	78.9%	113	21.1%	7.19 (0.007)
	Not improved	81	67.5%	39	32.5%	
District	Charsadda	235	83.9%	45	16.1%	63.5 (<0.01)
	Nowshera	172	62.3%	104	37.7%	
	DIK	97	97.0%	3	3.0%	
Toilet facility	Flush/pit latrine	361	79.3%	94	20.7%	5.26 (0.022)
	No facility	143	71.1%	58	28.9%	

**Table 2:** Prevalence of malnutrition in children of age 5-60 months based on underweight by gender.

	All *n* = 298	Boys *n* = 158	Girls *n* = 140
Prevalence of global malnutrition (<-2 *z*-score)	(75) 25.2% (20.6-30.4 95% CI)	(38) 24.1% (18.1-31.3 95% CI)	(37) 26.4% (19.8-34.3 95% CI)
Prevalence of moderate malnutrition (<-2 *z*-score and ≥-3 *z*-score)	(36) 12.1% (8.9-16.3 95% CI)	(21) 13.3% (8.9-19.5 95% CI)	(15) 10.7% (6.6-16.9 95% CI)
Prevalence of severe malnutrition (<-3 *z*-score)	(39) 13.1% (9.7-17.4 95% CI)	(17) 10.8% (6.8-16.6 95% CI)	(22) 15.7% (10.6-22.6 95% CI)

**Table 3:** Prevalence of acute malnutrition based on weight-for-age (underweight) *z*-score and by gender.

	All *n* = 656	Boys *n* = 371	Girls *n* = 285
Prevalence of global malnutrition (<-2 *z*-score)	(153) 23.3% (20.2-26.7, 95% C.I)	(81) 21.8% (17.5-26.3, 95% C.I.)	(72) 25.3% (20.6-30.6, 95%C.I.)
Prevalence of moderate malnutrition (<-2 *z*-score and ≥-3 *z*-score)	(108) 16.5% (13.8-19.5, 95%C.I.)	(60) 16.2% (12.8-20.3, 95% C.I.)	(48) 16.8% (12.9-21.6, 95%C.I.)
Prevalence of severe malnutrition (<-3 *z*-score)	(45) 6.9% (5.2-9.1, 95%C.I.)	(21) 5.7% (3.7-8.7, 95%C.I.)	(24) 8.4% (5.7-12.2, 95%C.I.)

**Table 4:** Bivariate logistic regression table for underweight among preschool and school-going children in the flood-affected area of Khyber Pakhtunkhwa.

Factors	Odds ratio	Std. err.	*z*	*p* > *z*	[95% Conf. Interval]	
					Lower	Upper
Sex of the child						
Male (r)	1	—	—	—	—	—
Female	0.9216108	0.2090305	-0.36	0.719	0.59	1.44
Child age						
1-12	6.916153	6.455428	2.07	0.038	1.11	13.09
12-24	5.839998	2.149672	4.79	<0.01	3.84	12.02
24-36	4.780962	2.171283	5.98	<0.01	2.62	12.70
>36 (r)	1	—	—	—	—	—
Maternal age						
15-24	2.893225	0.8597395	3.58	<0.01	1.62	5.18
25-34	1.380087	0.3823612	1.16	0.245	0.80	2.38
>34 (r)	1	—	—	—	—	—
Status of the HH						
IDP(r)	1	—	—	—	—	—
Host	1.803671	0.981647	1.08	0.278	0.62	5.24
Family type						
Individual family (r)	1	—	—	—	—	—
Joint family	1.244094	0.3063098	0.89	0.375	0.77	2.02
Family size						
Small (r)	1	—	—	—	—	—
Medium	1.365517	0.4075388	1.04	0.297	0.76	2.450972
Large	1.089051	0.3660427	0.25	0.800	0.56	2.10
Father occupation						
Not working	0.8523584	0.4186163	-0.33	0.745	0.32	2.23
Part time working	1.255285	0.6529761	0.44	0.662	0.45	3.48
Full time working (r)	1	—	—	—	—	—
Income						
5000-10000	1.078912	0.2980779	0.27	0.783	0.63	1.85
10000-15000	1.000419	0.2743983	-0.06	0.951	0.57	1.70
>15000 (r)	1	—	—	—	—	—
Mother education						
Literate (r)	1	—	—	—	—	—
Illiterate	1.388086	0.3309909	1.38	0.169	0.87	2.21
Father education						
Literate (r)	—	—	—	—	—	—
Illiterate	1.25752	0.3117022	0.92	0.355	0.77	2.04
Water quality						
Improved (r)	1	—	—	—	—	—
Not improved	3.404751	1.179312	3.54	<0.01	1.73	6.71
Toilet faculty						
Flush/pit latrine (r)	1	—	—	—	—	—
No facility	1.482691	0.3928416	1.49	0.137	0.88	2.49
District						
DIK (r)	1	—	—	—	—	—
Charsadda	3.58697	11.83281	4.26	<0.01	1.70	6.75
Nowshehra	5.44634	28.31439	5.62	<0.01	2.48	9.90
Cons	0.0013121	0.0012199	-7.14	<0.01	0.0002	.018

**Table 5:** Ordinal logistic regression model for undernutrition among preschool and school-going children in the affected area of Khyber Pakhtunkhwa.

Factors		Estimate	Std. error	Wald	Df	Sig.	95% confidence interval	
							Lower bound	Upper bound
Threshold	[Malnutrition status = 0.00]	1.362	0.761	3.202	1	0.074	-0.13	2.85
	[Malnutrition status = 1.00]	2.126	0.764	7.748	1	0.005	0.63	3.62
Location	Sex of the child							
	Male	-0.237	0.181	1.721	1	0.190	-0.59	0.12
	Female	1	.	.	0	.	.	.
	Child age (months)							
	0-12	2.157	0.901	5.731	1	0.017	0.39	3.92
	12-24	1.989	0.337	34.791	1	<0.01	1.33	2.65
	24-36	1.900	0.287	43.962	1	<0.01	1.34	2.46
	>36	1	-.	-.	0	.	.	.
	Maternal age							
	15-24	1.282	0.240	28.525	1	<0.01	0.81	1.75
	24-34	0.534	0.220	5.923	1	0.015	0.10	0.97
	Above 34	1	.	.	0	.	.	.
	Status of the HH							
	IDP's	0.174	0.389	.199	1	0.655	-0.59	0.94
	Host	1	.	.	0	.	.	.
	*Family type*							
	Joint family	.506	.197	6.593	1	0.010	0.12	0.89
	Individual family	1	.	.	0	.	.	.
	Family size							
	Small	-0.107	0.270	0.158	1	0.691	-0.64	0.42
	Medium	0.073	0.228	0.101	1	0.750	-0.37	0.52
	Large	1	.	.	0	.	.	.
	Father occupation							
	Not working	0.324	0.392	0.682	1	0.409	-0.45	1.10
	Part time working	-0.090	0.435	0.043	1	0.836	-0.94	0.76
	Full time working	1	.	.	0	.	.	.
	Mother occupation							
	Not working	0.892	0.575	2.409	1	0.121	-0.023	2.02
	Part time working	1.504	0.811	3.437	1	0.064	-0.09	3.09
	Not working	1	.	.	0	.	.	.
	Mother education							
	Literate	-0.342	0.192	3.173	1	0.045	-0.03	-0.71
	Illiterate	1	.	.	0	.	.	.
	Father education							
	Literate	-0.126	0.197	0.413	1	0.521	-0.51	0.26
	Illiterate	1	.	.	0	.		.
	Water quality							
	Improved	-0.839	0.261	10.363	1	0.001	-1.35	-0.33
	Not improved	1	.	.	0	.	.	.
	District							
	Charsadda	-0.077	0.298	0.067	1	0.796	-0.66	0.51
	Nowshehra	0.038	0.288	0.018	1	0.895	-0.53	0.60
	DIK	1	.	.	0	.	.	.
	Toilet facility							
	Flush/pit latrine	-0.939	0.199	22.308	1	<0.01	-1.33	-0.55
	No facility	1	.	.	0	.	.	.
	Income							
	5000-10000	0.096	0.23	0.172	1	0.67	-0.35	0.54
	10000-15000	0.073	0.21	0.113	1	0.73	-0.35	0.49
	>15000	1	.	.	0	.	.	.

## Data Availability

All data are included in this manuscript.

## Supplementary Materials

Supplementary Materials Figure S1 (a) shows the parentage of underweight and normal children in each age group and tells that there is a decreasing trend of malnutrition with the age of children because there is a high prevalence of stunting at low age groups as compared to higher age group (>36 months). Figure S1 (b) expresses the percentage of underweight and normal children for each age group calculated within the overall percentage of underweight and normal children in the target population. Figure S2 shows that out of total sample of size 656; 57.74% boys are with normal weights whereas 42.26% of girls are with normal weights. It is evident from the figure that male children are slightly more susceptible to underweight-based malnutrition than female children in the flood-affected areas of Khyber Pakhtunkhwa (KP). Figure S3 shows that out of total observed underweight children in the study area district, Nowshera is contributing the highest prevalence of underweight based malnutrition (68.42%) followed by district Charsadda (46.63%) and Dera Ismail Khan is the lowest (1.974% only). Figure S4 shows the pyramid for children age-stratified according to the districts. It has been shown that children belonging to district Nowshera were at high risk of underweight based undernutrition followed by district Charsadda also susceptible to undernutrition based on underweight compared to child belongs to Dera Ismail Khan. In the following Figure S5, the pyramid was constructed for children age-stratified according to gender. It showed that male children are slightly more susceptible to undernutrition based on underweight than female children.
