# Supplementary material for: Preparedness to Combat Determinants of Underweight-Based Child Malnutrition in Flood-Affected Areas of Pakistan
Source: Biomed Res Int. 2022 Jan 25;2022:6464901. doi: 10.1155/2022/6464901 (PMC8807031; doi:10.1155/2022/6464901)
Supplement: Supplementary Materials — Figure S1 (a) shows the parentage of underweight and normal children in each age group and tells that there is a decreasing trend of malnutrition with the age of children because there is a high prevalence of stunting at low age groups as compared to higher age group (>36 months). Figure S1 (b) expresses the percentage of underweight and normal children for each age group calculated within the overall percentage of underweight and normal children in the target population. Figure S2 shows that out of total sample of size 656; 57.74% boys are with normal weights whereas 42.26% of girls are with normal weights. It is evident from the figure that male children are slightly more susceptible to underweight-based malnutrition than female children in the flood-affected areas of Khyber Pakhtunkhwa (KP). Figure S3 shows that out of total observed underweight children in the study area district, Nowshera is contributing the highest prevalence of underweight based malnutrition (68.42%) followed by district Charsadda (46.63%) and Dera Ismail Khan is the lowest (1.974% only). Figure S4 shows the pyramid for children age-stratified according to the districts. It has been shown that children belonging to district Nowshera were at high risk of underweight based undernutrition followed by district Charsadda also susceptible to undernutrition based on underweight compared to child belongs to Dera Ismail Khan. In the following Figure S5, the pyramid was constructed for children age-stratified according to gender. It showed that male children are slightly more susceptible to undernutrition based on underweight than female children. [file 6464901.f1.docx]

**Supplementary files**

**These files has been cited in the main manuscript.**

**Description**

Figure S1 (a) shows the parentage of underweight and Normal children in each age group and tells that there is a decreasing trend of malnutrition with the age of children because there is a high prevalence of stunting at low age groups as compared to higher age group (>36 months). Figure S1 (b) expresses the percentage of underweight and normal children for each age group calculated within the overall percentage of underweight and normal children in the target population.

Figure S2 shows that out of total sample of size 656; 57.74% boys are with normal weights whereas 42.26% of girls are with normal weights. It is evident from the figure that male children are slightly more susceptible to underweight-based malnutrition than female children in the flood-affected areas of Khyber Pakhtunkhwa (KP).

Figure S3 shows that out of total observed underweight children in the study area district, Nowshera is contributing the highest prevalence of underweight based malnutrition (68.42%) followed by district Charsadda (46.63%) and Dera Ismail Khan is the lowest (1.974% only).

Figure S4 shows the pyramid for children age-stratified according to the districts. It has been shown that children belonging to district Nowshera were at high risk of underweight based under-nutrition followed by district Charsadda also susceptible to under-nutrition based on underweight compared to child belongs to Dera Ismail Khan.

In the following Figure S5, the pyramid was constructed for children age-stratified according to gender. It showed that male children are slightly more susceptible to under-nutrition based on underweight than female children.

**Figure S1**


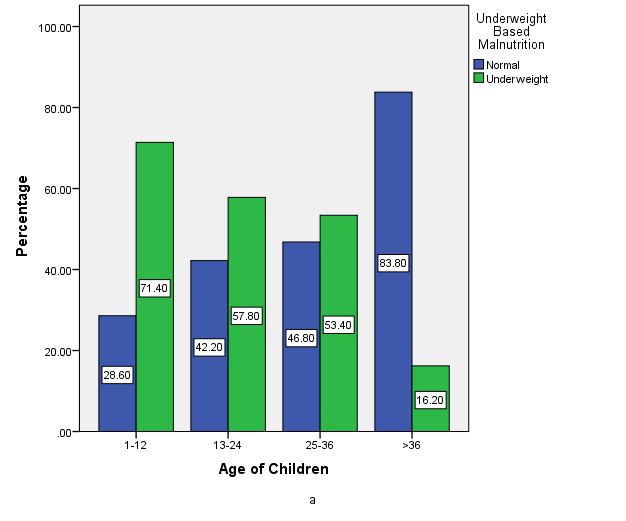

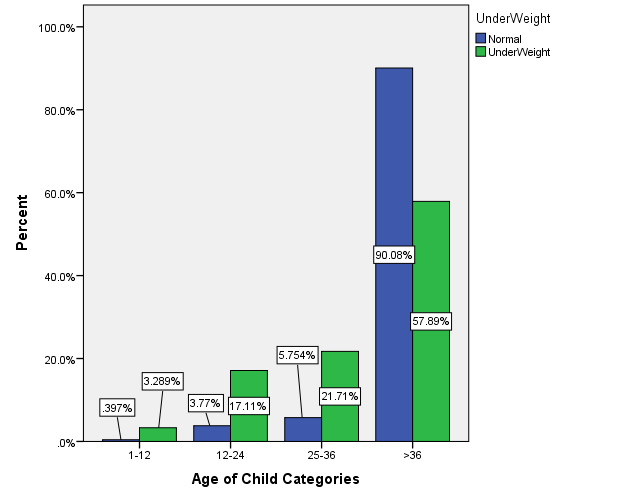


**Figure S1: (a & b): Age-wise Prevalence of Underweight Among Pre-school and School Going Children in Flood Hit Areas of KP**

**Figure S2**


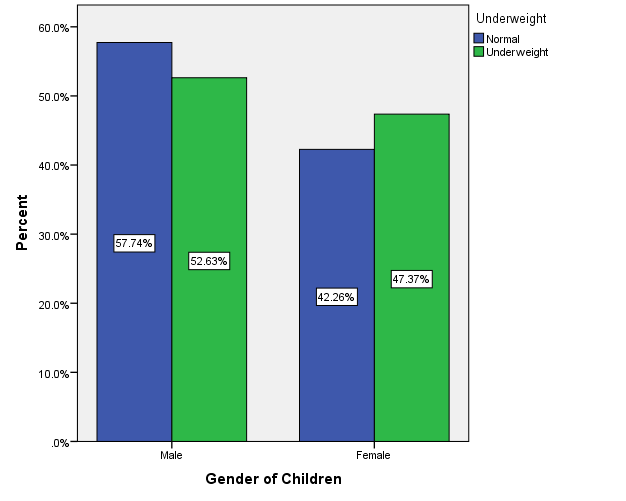


**Figure S2: Gender-wise Prevalence of Underweight Among Pre-school and School Going Children in Flood Hit Areas of KP**

**Figure S3**


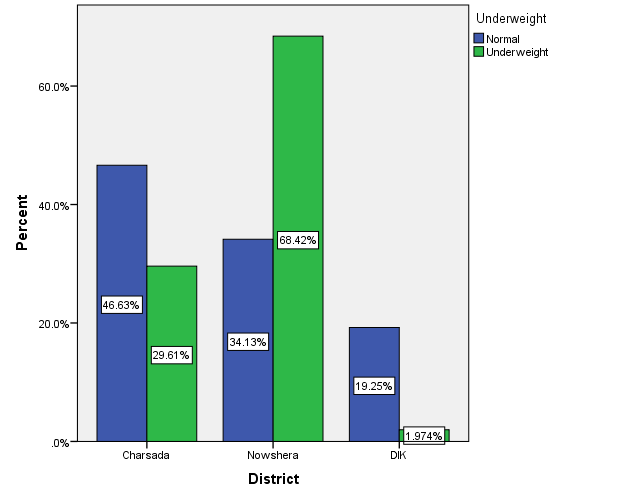


**Figure S3: District-wise Prevalence of Underweight Among Pre-school and School Going Children in Flood Hit Areas of KP**

**Figure S4**


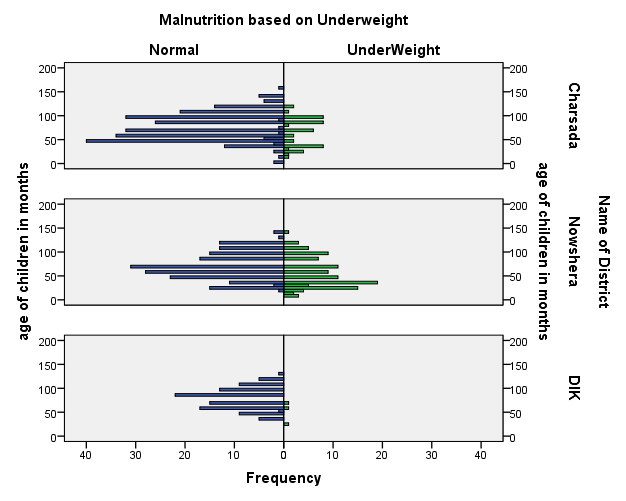


**Figure S4: Pyramid for malnutrition based on underweight for child age nested in three districts of flood hit areas of KP**

**Figure S5**


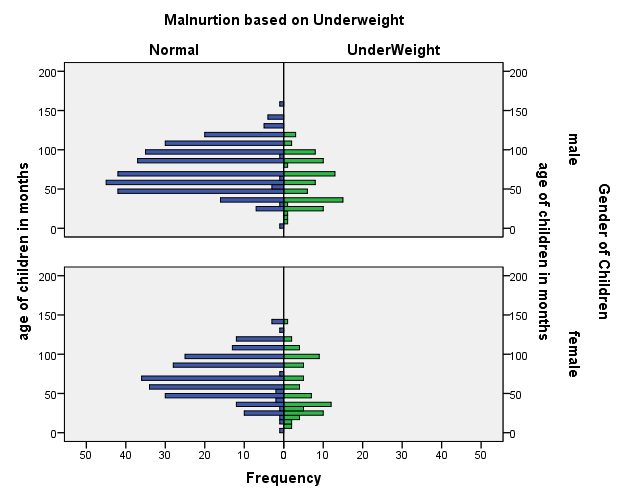


**Figure S5: Pyramid for malnutrition based on underweight for child age nested in gender of the pre-school and school going children.**
